# To Cure Stammering

**Published:** 1884-11

**Authors:** 


### To Cure Stammering.

Dr. Ralph Richardson writes to the *Brit. Med. Jour.*, Sept. 27, 1884, that any one may be cured of stammering by simply making an audible note in expiration before each word. Stammerers can sing as easily as other persons. Jacky Broster, of Chester, who made a large fortune by curing stammering, simply made his pupils say *her* before each word beginning with a consonant.

